# Disability Adjusted Life Years and minimal disease: application of a preference-based relevance criterion to rank enteric pathogens

**DOI:** 10.1186/1478-7954-6-7

**Published:** 2008-12-29

**Authors:** Juanita A Haagsma, Arie H Havelaar, Bas MF Janssen, Gouke J Bonsel

**Affiliations:** 1 Centre for Infectious Disease Control, National Institute of Public Health and the Environment, P.O. Box 1, Bilthoven, The Netherlands; 2 Institute of Health Policy and Management, Erasmus Medical Center, Erasmus University Rotterdam, P.O. Box 1738, Rotterdam, The Netherlands; 3 Institute for Risk Assessment Sciences, University of Utrecht, P.O. Box 80178, Utrecht, The Netherlands; 4 Department of Social Medicine, Academic Medical Center, University of Amsterdam, P.O. Box 22660, Amsterdam, The Netherlands

## Abstract

**Background:**

Burden of disease estimates, which combine mortality and morbidity into a single measure, are used increasingly for priority setting in disease control, prevention and surveillance. However, because there is no clear exclusion criterion for highly prevalent minimal disease in burden of disease studies its application may be restricted. The aim of this study was to apply a newly developed relevance criterion based on preferences of a population panel, and to compare burden of disease estimates of five foodborne pathogens calculated with and without application of this criterion.

**Methods:**

Preferences for twenty health states associated with foodborne disease were obtained from a population panel (n = 107) with the Visual Analogue Scale and the Time Trade-off (TTO) technique. The TTO preferences were used to derive the relevance criterion: if at least 50% of a panel of judges is willing to trade-off time in order to be restored to full health the health state is regarded as relevant, i.e. TTO median is greater than 0. Subsequently, the burden of disease of each of the five foodborne pathogens was calculated both with and without the relevance criterion.

**Results:**

The panel ranked the health states consistently. Of the twenty health states, three did not meet the preference-based relevance criterion. Application of the relevance criterion reduced the burden of disease estimate of all five foodborne pathogens. The reduction was especially significant for norovirus and rotavirus, decreasing with 94% and 78% respectively.

**Conclusion:**

Individual preferences elicited with the TTO from a population panel can be used to empirically derive a relevance criterion for burden of disease estimates. Application of this preference-based relevance criterion results in considerable changes in ranking of foodborne pathogens.

## Background

Since the application of the concept in 1993, the Disability Adjusted Life Year (DALY) is used increasingly for priority setting in disease control, prevention and surveillance [1]. The DALY is a health gap measure that aggregates mortality, acute morbidity and disability into a single index [2]. In order to combine information on mortality and morbidity, firstly the years lost due to premature mortality (YLL), and secondly the years lived with disability (YLD) have to be estimated. The latter result from a computational procedure that combines duration and severity of both acute disease and disability from sequelae.

An essential component of YLD computation is the disability weight. A disability weight is a scaling factor assigned to living with disability that ranges from 0 (best possible health state) to 1 (worst possible health state or equating death) [3]. This value reflects the impact of the disability on the health-related quality of life and is commonly based on the preferences of a panel of judges [4].

To arrive at YLDs, the disability weights have to be combined with incidence and duration data. In some burden of disease studies hospital admissions and Emergency Department treatments are used as data source in this regard [5,6]. Other studies use data from General Practitioner registries or population health surveys [7,8]. Although the latter approach yields otherwise lacking incidence data which are vital to YLD calculations, such registries and surveys have low response thresholds, implying that cases of minimal disease are also included.

Minimal disease consists of temporary health states of short duration that have an anticipated and observed minor impact on individual health-related quality of life. However, due to high prevalence of most minimal disease, collectively they may account for a large number of YLDs in the aggregate, and may therefore get policy priority above severe, but less frequently occurring diseases. As a result, the application of burden of disease estimates in prioritization discussion may be obfuscated. This can be overcome by including a criterion for relevant disease. A prerequisite of this relevance criterion is that it is able to distinguish 'experienced' minimal disease from relevant disease unambiguously, yet it should also allow relevant mild disease to be included in burden of disease estimates in order to avoid incomplete estimates of diseases characterized by heterogeneous levels of severity.

Other than on health care use, the criterion to distinguish minimal diseases from relevant diseases may be based on health outcome; anatomical characteristics of the disease, absenteeism (work, school), or on societal preferences that are derived to assess the disability weights necessary for the YLD calculation. To obtain the latter, preferences from a panel of judges elicited with dedicated preference measurement methods commonly are used [9,10]. A preference measurement method widely used in these panel studies is the time trade-off (TTO) method. The TTO method requires an individual to give up time in order to be restored from the health state to full health [10]. The more time the participant is willing to offer, the less desirable the health state is compared with perfect health. In the Dutch Mild Diseases and Ailments study, Bonsel et al. developed a relevance criterion based on the TTO preferences to distinguish relevant from minimal conditions [11]. The preference-based relevance criterion is met if the median TTO value is greater than 0, i.e. if at least 50% of a panel of judges is willing to trade-off time in order to be restored to full health. If not, the health state is regarded as not relevant and excluded from the burden of disease calculation. The cut-off point of the criterion, where at least 50% of the panel member has to be willing to trade-off time, corresponds to the majority rules principle of most democratic voting systems.

In the area of foodborne disease, often high incidences of infectious disease are observed with most cases leading to full recovery within only a few days. Comprehensive studies on foodborne disease are vulnerable to inadvertently putting too much emphasis on such minimal diseases.

The aim of our study in this context was 1) to derive a relevance criterion for foodborne disease based on the preferences of a population panel, and 2) to compare YLD estimations of five common foodborne pathogens calculated with and without the application of the preference-based relevance criterion.

## Methods

### Health state description

Five enteric pathogens were selected, namely norovirus and rotavirus, thermophilic *Campylobacter *spp., *Salmonella *spp., and Shiga-toxin producing *Escherichia coli *O157 (STEC O157). We carefully defined the diseases caused by these foodborne pathogens, collected empirical evidence on the associated functional consequences over time and subdivided the diseases into disease severity grades that were presumed to be homogeneous regarding disability, treatment and prognosis. This resulted in 20 health states.

The functional consequences of each of the 20 health states were presented on a vignette. A vignette is a preformatted A4 size sheet that provided the disease label, clinical description and a generic description. For the generic description, we used an extended version of the EQ-5D classification system [12-14]. This classification system describes health with five levels of severity in the dimensions mobility, self-care, usual activities, pain/discomfort, anxiety and depression and cognition [15].

Additionally, the vignette provided a visual representation of the body sites affected by the disease, and described the duration of the disease over one year time. The duration of the condition was presented as an annual profile, which describes the course of the health state – the disability profile – over one year, allowing assessment of diseases with a rapid course [16,17]. Conditions with short duration were presented as a patient who in an otherwise healthy year experiences, for instance, the health impact of mild gastroenteritis for the duration of one week; whereas conditions with long term consequences were presented as a patient who experiences, for instance, the consequences of Guillain-Barré syndrome throughout the whole year.

### Health state valuation

The Visual Analogue Scale (VAS) and the TTO were used to elicit preferences for the 20 health states [18]. The VAS valuation technique requires participants to score the health state on a vertical rating scale graded from 0 (worst imaginable health state) to 100 (best imaginable health state). In the TTO the participants were asked how many days of one year in full health they were willing to trade in order to be restored from the presented disease stage to full health. A trade-off of time implies a shorter life expectancy in exchange for full health during the remaining time. The participants were instructed to contemplate the year described by the vignette only, and to ignore any prognostic element of what could happen after one year.

All health states were valued independently according to both methods.

### Participants and data-collection

The panel participants were randomly selected from a sample of 560 lay people who applied to participate in the Mild Diseases and Ailments Study, conducted in 2003 [17]. For the Mild Diseases and Ailments Study people were recruited from the general public via an advertisement in a newspaper that is freely available throughout the Netherlands. For this study, a random sample of 150 persons was drawn and contacted by mail of which 115 were willing to participate.

Valuation data were collected through a two-step procedure. Firstly, the participants attended a three hour panel session, during which they valued 10 vignettes with the VAS and the TTO. The second part of the data-collection consisted of an unsupported postal questionnaire which the participants received at home one week after attending the panel meeting. Apart from the 20 health states related to foodborne disease, the participants valued 24 other health states (core health states inserted to verify reliability among groups, work-related health states and psychiatric health states) totalling the number of questionnaire vignettes to 34. Because of the high total number of vignettes, we developed two versions of the questionnaire. Each version of the questionnaire asked the participant to value 17 vignettes with the VAS and the TTO. The order of the vignettes was randomized and the questionnaire version was randomly assigned to the participants. The participants received 20 euros for attending the panel session and 30 euros for filling out the follow up questionnaire.

### Data-analysis

Firstly, we standardized VAS and TTO weights for each individual response using the following formulas:

VAS weight = 1 - (VAS score/100)

TTO weight = TTO score/365

To establish whether the disease stages were ranked in consistent order, the ranking of a vignette according to the VAS and TTO weights was compared using Spearman's and Pearson's rank correlation coefficients. We performed regression analysis to determine whether socio-demographic variables age, sex, education level and disease experience had independent significant effects on the VAS and TTO weights. To establish the inter-rater reliability, which measures group homogeneity, we defined each of the eight panel meetings as a group and calculated the intra-class correlation (ICC) of these groups.

In order to calculate the YLD, national incidence data on foodborne disease (year 2004) was combined with the disability weight derived from the panel study. YLD was calculated both with and without application of the relevance criterion. The relevance criterion implies that if the median TTO = 0, the disease stage is regarded as not relevant and therefore not included in the YLD calculation.

To calculate YLL we used mortality data from Statistics Netherlands. The resulting YLL were combined with YLD in order to calculate the number of DALYs lost due to the five foodborne pathogens, all following standard procedures (no age weighting). For a more detailed description of the DALY calculation, see Kemmeren et al[19].

## Results

### Participants

The panel meeting was attended by 107 participants. Each of the 107 participants responded to the questionnaire. On average it took the participants 1 hour and 43 minutes to fill out the questionnaire. The average age of the participants was 51 years and 62% was female. Statistics of the participants are presented in Table 1.

**Table 1:** Statistics of the population panel

**Statistics**	
Age (in years)	51.2 [21–79]
	
Sex	
Male	41 (38%)
Female	66 (62%)
	
Educational level	
Low	22 (21%)
Middle	43 (40%)
High	41 (38%)
Unknown	1 (1%)
	
Disease experience	
Yes	55 (51%)
No	51 (48%)
Unknown	1 (1%)

### Health state valuations

Table 2 presents the mean and median TTO weights for the 20 health states. Mild conditions with short duration, like gastro-enteritis, was rated lowest (mean TTO weight 0.01) whereas severe long-term disease, like the Guillain-Barré syndrome level F5, was rated highest (mean TTO weight 0.46). Weights increased by level of severity within the diseases; weights attributed to mild reactive arthritis (mean TTO weight 0.02) were lower than moderate reactive arthritis (mean TTO weight 0.12) and severe reactive arthritis (mean TTO weight 0.19) respectively. The percentage of participants that were not willing to trade-off any time decreased by the level of severity within the disease; for moderate hemolytic uremic syndrome 13% of the participants were not willing to trade-off time, whereas for severe hemolytic uremic syndrome this was 0%. The standard deviation of the TTO values was higher in the middle range, which was anticipated given the fixed end points of the scale. Correlation coefficients of VAS and TTO values were high, Pearson's correlation coefficient was 0.92 and Spearman's correlation coefficient was 0.95. ICC, which indicates the inter-rater reliability, was 0.99 for the VAS values and 0.97 for the TTO values. No significant effects of age, sex, and disease experience on TTO values were demonstrated. Educational level, however, did have a significant effect on the TTO values of the Guillain-Barré disease stages.

**Table 2:** Mean VAS weights and mean and median TTO weights, per health state

**Health state and length of illness**		**VAS**	**TTO**			
				
	**n**	**mean**	**median**	**mean**	**sd**	**%0^a^**
*Gastroenteritis*						
Gastroenteritis, mild, 1 day	51	0.036	0	0.002	0.01	88
Gastroenteritis, mild, 5 days	53	0.102	0	0.010	0.04	60
Gastroenteritis, moderate, 10 days	107	0.130	0.005	0.015	0.04	26
Gastroenteritis, severe, 7 days	53	0.231	0.008	0.025	0.05	25
Gastroenteritis, severe, 14 days	51	0.295	0.011	0.041	0.07	17
Gastroenteritis, chronic, 6 months	53	0.368	0.058	0.099	0.11	8
						
*Guillain-Barré syndrome *^b^						
Guillain-Barré syndrome, F1, whole year	51	0.185	0.008	0.044	0.09	40
Guillain-Barré syndrome, F2, whole year	107	0.420	0.077	0.137	0.18	7
Guillain-Barré syndrome, F3, whole year	53	0.545	0.153	0.215	0.23	2
Guillain-Barré syndrome, F4, whole year	51	0.700	0.252	0.367	0.32	2
Guillain-Barré syndrome, F5, whole year	53	0.722	0.403	0.460	0.31	0
						
*Reactive arthritis*						
Reactive arthritis, mild, 1 week	51	0.107	0	0.004	0.01	68
Reactive arthritis, mild, 6 weeks	53	0.197	0.011	0.023	0.03	25
Reactive arthritis, moderate, 6 months	53	0.447	0.058	0.115	0.13	8
Reactive arthritis, severe, 6 months	51	0.503	0.153	0.186	0.16	4
						
*Hemolytic Uremic Syndrome (HUS)*						
HUS, moderate, 1 month	53	0.279	0.022	0.056	0.08	13
HUS, severe, 1 month	51	0.481	0.038	0.110	0.19	0
Renal failure, whole year	51	0.628	0.252	0.328	0.25	0
						
*Inflammatory bowel disease*						
Crohn disease, 6 months	51	0.347	0.067	0.105	0.12	4
Colitis ulcerosa, 6 months	53	0.492	0.115	0.154	0.15	7

^a ^percentage of participants that were not willing to trade-off any time in order to be restored from the health state.

^b ^for a detailed description of the five health states of patients with Guillain-Barré syndrome, see Havelaar et al. [8]

### Preference-based relevance criterion

Table 2 shows that three health states, namely mild gastro-enteritis with a length-of-illness of respectively one day, and one week, and mild reactive arthritis with length-of-illness of one week, had a TTO median of 0. Therefore, these three disease stages did not meet the relevance criterion.

Table 3 presents the TTO weights elicited in two previous Dutch health state valuation studies conducted in 2003 and 2005 that had a design similar to the current study [17,20]. The results show that three health states, namely common cold with length-of-illness of one week, onychomycosis with a length-of-illness of 52 weeks, and superficial injury with a length-of-illness of four weeks had a TTO median of 0 in both studies.

**Table 3:** Median TTO weights of 10 health states valued in two preceding panel studies, by health state

	**2003**			**2005**		
		
**Health state**	**n**	**TTO median**	**%0^a^**	**n**	**TTO median**	**%0^a^**
Common cold, 7 days	101	0	85	140	0	81
Cystitis, 2 weeks	102	0.003	47	64	0.005	27
Rhinitis, 17 weeks	102	0.003	38	64	0.019	28
Eczema, whole year	102	0.019	16	64	0.044	11
Gastritis, 4 weeks	101	0.005	37	64	0.019	16
Onychomycosis, whole year	102	0	83	63	0	60
Osteoporosis, whole year	102	0.003	45	142	0.003	36
Otitis, 2 weeks	101	0.003	40	64	0.010	44
Superficial injury, 4 weeks	32	0	53	142	0	72
Open wound, 4 weeks	34	0.005	41	47	0.003	49

^a ^%0 = the percentage of participants that were not willing to trade-off any time in order to be restored from the health state

### DALY calculation

The burden of disease was calculated for all five foodborne pathogens (see Table 4). Without application of a relevance criterion most DALYs were lost due to norovirus (2940 DALYs), rotavirus (1327 DALYs) and thermophilic *Campylobacter *spp. (1137 DALYs). Least DALYs were lost due to *Salmonella *spp. (747 DALYs) and STEC O157 (120 DALYs).

**Table 4:** Incidence and disease burden calculated with and without the preference-based relevance criterion (RC), by pathogen

			***Without RC***		***With RC***	
			
**Pathogen**	**Incidence**	**YLL**	**YLD**	**DALY**	**YLD**	**DALY**
**Norovirus**						
Gastroenteritis	472,000	55	2885	2940	121	175
						
**Rotavirus**						
Gastroenteritis	190,000	110	1217	1327	176	287
						
**Thermophilic *Campylobacter *spp**.						
Gastroenteritis	59,400	390	420	810	148	538
Guillain-Barré syndrome	60	35	150	185	150	185
Reactive arthritis	864	-	40	40	40	40
Inflammatory Bowel Disease	22	-	102	102	102	102
Total				1137		865
						
***Salmonella *spp**.						
Gastroenteritis	35,400	440	255	697	77	517
Reactive arthritis	460	-	17	17	17	17
Inflammatory Bowel Disease	7	-	33	33	33	33
Total				747		567
						
**STEC O157**						
Gastroenteritis	1,300	6	13	19	7	13
Hemolytic Uremic Syndrome	20	76	25	101	25	101
Total				120		114

Application of the relevance criterion resulted in a burden of disease estimate of 175 DALYs lost due to norovirus and 287 DALYs lost due to rotavirus, a decrease of 94% and 84%, respectively. For thermophilic *Campylobacter *spp. the burden of disease reduced by 24% to 865 DALYs, and for *Salmonella *spp. by 24% to 567 DALYs. With 5%, the decrease in burden of disease was smallest for STEC O157, which reduced from 120 to 114 DALYs.

As a result of the reductions in part of the burden of disease estimates, the ranking of the foodborne pathogens changed. Without a relevance criterion, the ranking according to descending burden of disease was: 1) norovirus, 2) rotavirus, 3) thermophilic *Campylobacter *spp., 4) *Salmonella *spp., and 5) STEC O157. Conversely, when the preference-based relevance criterion was applied the ranking was: 1) thermophilic *Campylobacter *spp., 2) *Salmonella *spp. 3) rotavirus, 4) norovirus, and 5) STEC O157.

## Discussion

The results showed that for three health states associated with foodborne disease less than 50% of the panel members were willing to trade-off any time. Therefore these health states did not meet the relevance criterion proposed by Bonsel et al. [11]. Application of the preference-based relevance criterion reduced the burden of disease estimates of all five foodborne pathogens, varying from 94% (norovirus) to 5% (STEC O157). The ranking of the foodborne pathogens changed considerably when the relevance criterion was applied.

The burden of disease would decrease even more when disability weights based on median rather than mean TTO values were used to calculate YLD. The benefit of using median TTO values is that the majority rules principle is applied to all health states and not only minimal disease.

In our study, the three health states that did not meet the preference-based relevance criterion of median TTO > 0 all had length-of-illness of one week or less and this short duration may have resulted in a majority of participants not willing to trade-off any time. Nonetheless, the results of two similar health state valuation studies showed that several health states that lasted over one week did not meet the preference-based relevance criterion either [17,20]. Of the twelve health states that had a median TTO of 0, eight had a length-of-illness of over one week, varying from two weeks (genital candidiasis female) through 52 weeks (onychomicosis), whereas health states with a length-of-illness of one week or less on the other hand did meet the criterion [17]. This indicates that both duration and symptom severity matter for the panel of judges and that health state valuation studies are necessary to determine which health state meet the TTO median > 0 criterion.

In the Netherlands, intestinal infectious disease ranks among the least burdening conditions and the results of this study might give the impression that application of the relevance criterion will substantially diminish it even further compared to other diseases [7]. However, it should be noted that of the many pathogens causing foodborne disease the current study addressed five. Not only is there a difference in disease caused by the foodborne pathogens, the severity and duration of the disease is also highly dependent of the condition of the patient. So for each cause of disease, foodborne or other, it should be carefully considered which health states are caused by the pathogen and whether all patients meet the health state descriptions.

Previously, relevance criteria based on anatomy and health care use have been suggested [21]. A problem with anatomical criteria is, however, that they cannot be applied to every condition and that for each group of diseases a specific anatomical criterion has to be formulated by experts. On the other hand, criteria based on health care use may be affected by differences in access to health care, resulting in incomparable disease burden [22]. Moreover, both anatomical and health care use criteria might be stringent and not allow relevant mild diseases to be included in burden of disease calculations. This might result in incomplete burden of disease estimates, an issue that is especially of importance for conditions characterized by heterogeneous levels of severity like foodborne disease and injury. This is underscored by the results of a recent study that showed a 36% increase in burden of disease if relevant mild injuries were included in burden on injury calculations [20].

The aforementioned problems are overcome by the preference-based relevance criterion. This method to empirically derive a relevance criterion is simple and transparent, and the resulting relevance criterion can be applied to each condition. Moreover, the relevance criterion is based on individual preferences derived from a population panel, which concurs with the societal perspective of the burden of disease concept [23]. Furthermore, as shown by the results of this study, the preference-based relevance criterion is sensitive for relevant mild disease.

A limitation of the preference-based criterion is that for any new health state preferences have to be obtained in order to derive the preference-based criterion. On the other hand, the results of the current study point out that preferences obtained from new panel studies collate with preferences derived previously with the same protocol.

Furthermore, it should be noted that the TTO preferences from this study are derived with the annual profile approach in stead of the standard QALY/DALY approach used in the original DALY-approach of the Global Burden of Disease study [3]. Unlike the annual profile approach, the standard QALY/DALY approach assumes independence between duration and disability and requires that the health state remain fixed over time. This means that in order to assess preference weights health states with an acute onset, episodic diseases like epilepsy, and health states characterized by complex and heterogeneous recovery patterns, have to be separated into numerous parts.

To alleviate this inability to assess preference weights for health states with complex patterns of duration and severity, the annual profile approach was developed [4]. The most important feature of the annual profile approach is that the course of the health state is described over one year time. The results of current and previous studies demonstrated that the annual profile approach yields valid and reliable disability weights for stable health states as well as health states that vary widely over time [13,17,20]. In absence of an algorithm to transform scores into utility values, the annual profile requires new panel data when fresh disease states have to be valued. Yet, this does not imply that an infinite amount of states needs to be valued. The actual number required depends on the observed variability of severity-duration combinations. In most diseases less than ten states will suffice to cover the known heterogeneity of the disease. A second criticism of the annual profile approach was that it would overvalue diseases with a rapid course [24]. According to this criticism, application of these disability weights might overestimate the burden of disease. In their turn, Essink-Bot & Bonsel pointed out that this overvaluation of health states are a result of discrimination between low severity conditions rather than time presentation [4].

This alleged lack of discrimination between low severity conditions is not endorsed by the results of the current study, which show that the population panel in the Netherlands assigned values to health states that including the low severity conditions increase by level of severity of the health state. Additionally, they appeared to be capable to discriminate minimal from relevant disease and the preference-based relevance criterion appeared to be stable over time. Moreover, Janssen et al. (2008) showed that the criterion is similar for lay people, medical advisors, as well as general practitioners [17].

Conversely to the agreement between panels with different perspectives, it is not yet clear whether the preference-based relevance criterion is similar for different countries. Diseases that did not meet the relevance criterion in this study could be regarded as relevant by people who do not have good access to good sanitary facilities and health care. Previous studies did find that ranking of health states is similar across countries, yet the assigned values differed significantly [16,25,26]. Since the preference-based relevance criterion is based on values rather than ranking, differences concerning the criterion are expected.

Secondly, it remains to be investigated whether the approach used to derive the preferences affects the preference-based relevance criterion. In the current study, the annual profile approach was used. The alternative standard QALY/DALY approach, unlike the annual profile approach, presents diseases with a so-called period profile. Using the standard QALY/DALY approach to derive preferences may cause a shift in the derived preferences and consequently affect the preference-based relevance criterion.

## Conclusion

We conclude that individual preferences derived with the TTO method from a population panel can be used to empirically derive a relevance criterion for burden of disease estimates, and that application of this preference-based relevance criterion results in considerable changes in ranking of foodborne pathogens.

## Abbreviations

DALY: Disability Adjusted Life Year; HUS: Hemolytic Uremic Syndrome; STEC O157: Shiga-toxin producing *Escherichia coli *O157; TTO: Time Trade-off; VAS: Visual Analogue Scale; YLD: Years Lived with Disability; YLL: Years of Life Lost.

## Competing interests

The authors declare that they have no competing interests.

## Authors' contributions

JH constructed the disease vignettes, performed the panel study, executed the statistical analysis and drafted the manuscript. AH supervised the construction of the vignettes, collected population data necessary for the burden of disease calculation and assisted with the drafting the manuscript. MJ participated in the design and execution of the panel study. GB supervised and participated in the design of the panel study and drafting of the manuscript.
